# Real-time 3D US–CT fusion-based semi-automatic puncture robot system: clinical evaluation

**DOI:** 10.1007/s11548-025-03489-9

**Published:** 2025-08-05

**Authors:** Masayuki Nakayama, Bo Zhang, Ryoko Kuromatsu, Masahito Nakano, Yu Noda, Takumi Kawaguchi, Qiang Li, Yuji Maekawa, Masakatsu G. Fujie, Shigeki Sugano

**Affiliations:** 1 Graduate School of Creative Science and Engineering, Waseda University, Tokyo, 1698555 Japan; 2 Future Robotics Organization, Waseda University, Tokyo, 1620044 Japan; 3 Faculty of Science and Engineering, Waseda University, Tokyo, 1698555 Japan; 4 Division of Gastroenterology, Department of Medicine, Kurume University School of Medicine, Fukuoka, 8300011 Japan; 5 KYOSETO Co., Ltd, Tokyo, 1600023 Japan

**Keywords:** Surgical robot, Robotic puncture system, Fusion imaging, Blood vessel segmentation

## Abstract

**Purpose:**

Conventional systems supporting percutaneous radiofrequency ablation (PRFA) have faced difficulties in ensuring safe and accurate puncture due to issues inherent to the medical images used and organ displacement caused by patients’ respiration. To address this problem, this study proposes a semi-automatic puncture robot system that integrates real-time ultrasound (US) images with computed tomography (CT) images. The purpose of this paper is to evaluate the system's usefulness through a pilot clinical experiment involving participants.

**Methods:**

For the clinical experiment using the proposed system, an improved U-net model based on fivefold cross-validation was constructed. Following the workflow of the proposed system, the model was trained using US images acquired from patients with robotic arms. The average Dice coefficient for the entire validation dataset was confirmed to be 0.87. Therefore, the model was implemented in the robotic system and applied to clinical experiment.

**Results:**

A clinical experiment was conducted using the robotic system equipped with the developed AI model on five adult male and female participants. The centroid distances between the point clouds from each modality were evaluated in the 3D US–CT fusion process, assuming the blood vessel centerline represents the overall structural position. The results of the centroid distances showed a minimum value of 0.38 mm, a maximum value of 4.81 mm, and an average of 1.97 mm.

**Conclusion:**

Although the five participants had different CP classifications and the derived US images exhibited individual variability, all centroid distances satisfied the ablation margin of 5.00 mm considered in PRFA, suggesting the potential accuracy and utility of the robotic system for puncture navigation. Additionally, the results suggested the potential generalization performance of the AI model trained with data acquired according to the robotic system's workflow.

## Introduction

PRFA has become widely adopted as a minimally invasive treatment for liver cancer. While PRFA imposes a lower burden on patients compared to surgical procedures, physicians are required to navigate the puncture path planned preoperatively and accurately puncture the hepatic tumor using US images. Although US imaging provides the advantages of real-time visualization and the absence of radiation exposure, it presents challenges such as noise and low contrast between soft tissues. Furthermore, internal organs may shift due to respiratory movement [1]. Physicians are required to have substantial experience and advanced technical skills to ensure precise puncture.

To assist in safe puncture procedures, methods for detecting needles and target blood vessels in US images have been proposed [2, 3]. Additionally, studies on fusion systems that integrate preoperative CT and US images to provide more detailed information have also been conducted [4]. Despite these advancements, significant burdens associated with puncture planning and exploration remain. Consequently, various robotic systems for puncture procedures have been developed to directly support physicians' techniques. For example, studies have been conducted on performing robotic puncture using information from optical markers placed on the patient’s body surface during CT imaging [5]. However, this approach is limited in its applicability and raises concerns about radiation exposure for patients who have already had CT imaging completed. Recent studies have examined robotic arm systems that automatically control US probes by utilizing deep reinforcement learning to mimic physicians' probe scanning and identify optimal US cross-sections [6]. This approach enhances versatility by eliminating the need for external sensors other than the US probe. Nevertheless, exclusive reliance on US images makes it difficult to capture detailed anatomical information and necessitates substantial preoperative data to compensate for individual variations and respiratory-induced organ movement. To address respiratory movement, algorithms that track respiratory cycles using diaphragm motion have been developed, enabling tumor tracking without additional sensors [7]. These algorithms alone do not support the tracking of all organs and blood vessels, making it challenging to establish a precise and safe puncture path. Furthermore, maintaining accurate respiratory tracking under the constraints of rib-induced motion limitations remains technically challenging. Other robotic systems have been proposed to compensate for movement by synchronizing with the patient’s motion through attachment to the patient’s body surface [8]. Although the number of scans can be reduced, multiple CT scans are still required during puncture procedures, which remains a concern.

To address these issues, we propose a novel robotic puncture assistance system that integrates real-time US images with detailed anatomical information from CT images through 3D US–CT fusion [9]. In evaluation experiments involving an abdominal phantom and live pigs under ventilator-controlled respiration, the system demonstrated compliance with the maximum ablation margin of 5.00 mm [10], as defined in PRFA procedures. However, in clinical settings involving actual patients, PRFA is typically performed by instructing the patient to temporarily hold their breath during natural respiration. The primary objective of this study is to evaluate the feasibility and efficacy of the semi-automated puncture assistance robotic system using real-time US images in its pilot clinical experiment involving patients.

In this study, the workflow of the proposed robotic system is first outlined. Next, an AI-based blood vessel recognition model was developed using patient US images to prepare for the clinical experiment. Subsequently, an evaluation experiment of the 3D US–CT fusion system based on the constructed AI model was conducted. Finally, the clinical utility of the robotic puncture assistance system is discussed.

## Methods

### System design

**Fig. 1:**
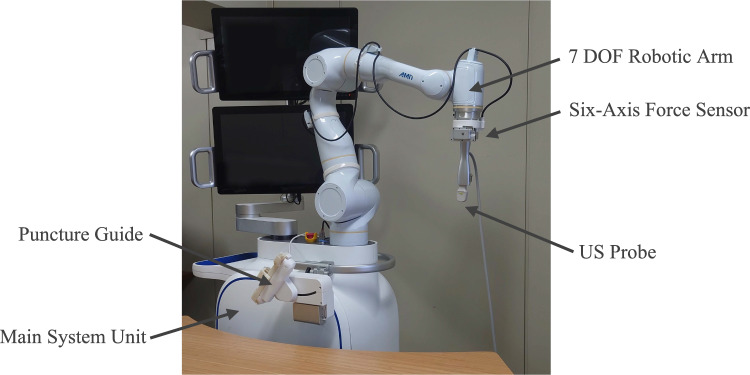
The overview of the puncture robot system

**Fig. 2:**
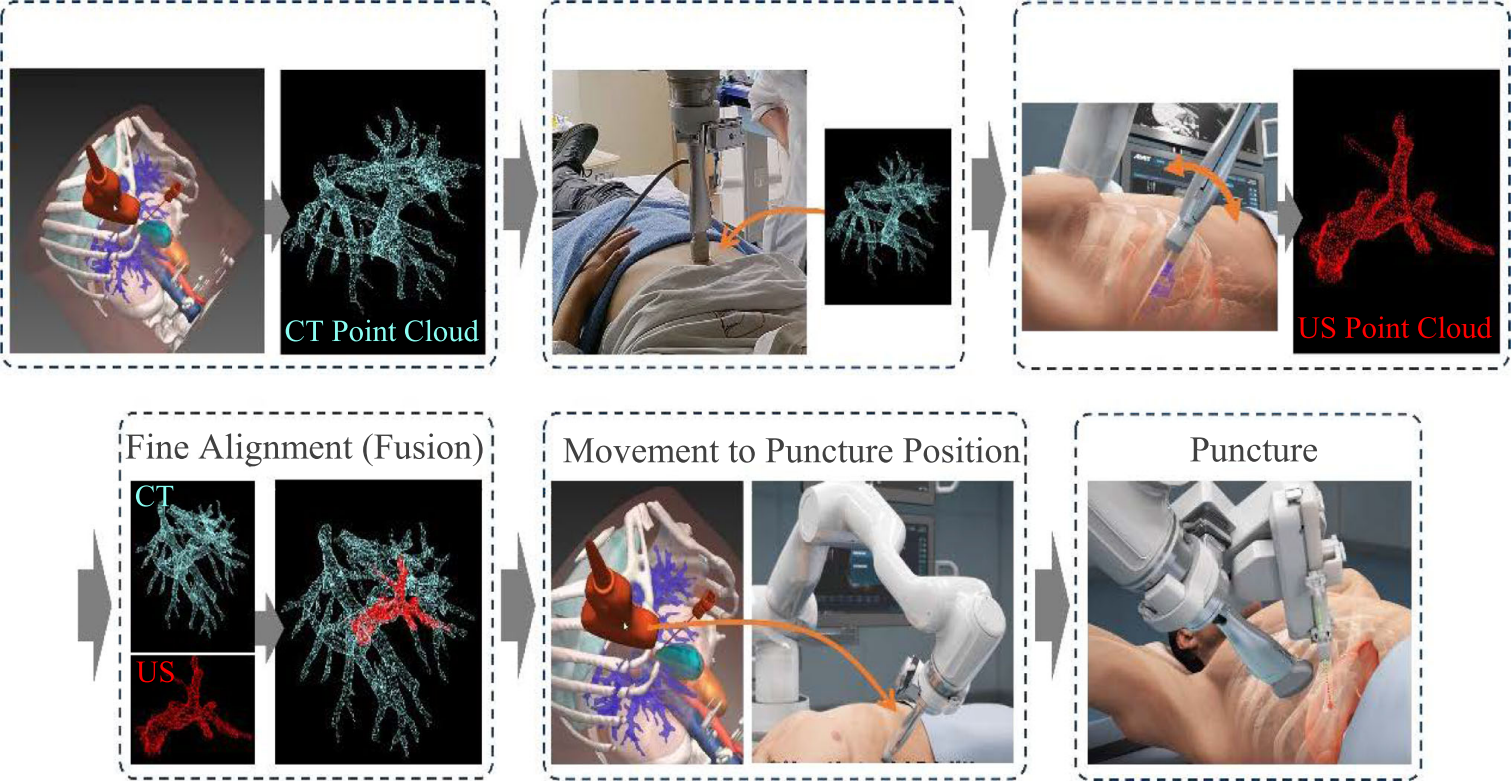
The workflow of the puncture navigation using 3D US–CT fusion

The semi-automated robotic puncture assistance system used in this study is shown in Fig. 1. As described in the previous research, the system comprises a 7 degree-of-freedom (DOF) robotic arm equipped with a US probe. Under the supervision of a physician, the robotic arm performs automated probe scanning and assists with puncture procedures. A six-axis force sensor is connected to the base of the US probe to ensure patient safety, and the system is configured to perform an emergency stop if the contact force exceeds 30 N. The system consists of two primary components: preoperative planning and intraoperative navigation. The workflow for operating the system is divided into the following six steps, as shown in Fig. 2.

1. **Preoperative Planning:** Using preoperative planning software, a 3D anatomical model of the abdomen and a 3D CT point cloud of the intrahepatic blood vessels are constructed based on contrast-enhanced CT images. Physicians can intuitively define a safe puncture path on the 3D model. The planned puncture path and the 3D CT point cloud of the intrahepatic vessels are utilized during intraoperative navigation.
2. **Coarse Alignment of the Robotic System:** In the initial step of intraoperative navigation, the robotic arm is positioned by the physician at an anatomical landmark located along the midline of the body. The xiphoid process is recommended as the reference point. Once positioned, the robotic system automatically aligns the CT coordinate system with the robotic arm’s coordinate system to correspond with the landmark and probe position.
3. **Preparation for Fine Alignment of the Robotic System:** The probe is positioned by the physician at an intercostal position where intrahepatic blood vessels are clearly visible. One of the major strengths of our system is that it does not rely on specific anatomical landmarks such as tumors, and scanning any intrahepatic vessel is sufficient. This is because the fusion between US and CT is achieved by using geometry of blood vessels, which is an anatomical feature of the human body. This approach allows the physician to select intrahepatic vessels with high visibility and perform the scan, which can be particularly beneficial for inexperienced physicians who may find it difficult to capture specific anatomical structures. After instructing the patient to hold their breath, the physician activates the foot switch to initiate automated scanning by the robotic arm. The robotic arm scans the probe in a sweeping motion, tilting the US imaging plane by ± 15 deg relative to the probe's tip over approximately 5 s.
4. **3D US–CT Fusion (Fine Alignment):** The US images acquired during the scanning process are transmitted to the system, which constructs a 3D US point cloud of blood vessels. Subsequently, a feature-based registration method using an improved Iterative Closest Point (ICP) algorithm is employed to align the 3D CT point cloud with the US point cloud. Since the coordinate systems of each modality have already been brought closer by the coarse registration, the risk of converging to a global–local minimum is reduced.
5. **Movement to the Puncture Position:** Based on the results of the 3D US–CT fusion, the system automatically calculates the transition path from the current fan scan position to the puncture path location defined in the preoperative plan. The physician activates the foot switch, and the robotic arm automatically moves to the puncture path location.
6. **Puncture Procedure:** The puncture guide mechanism is attached to the robotic arm, and the physician manually inserts the needle while monitoring the real-time US images.

### Development of AI for blood vessel recognition in ultrasound images

**Fig. 3:**
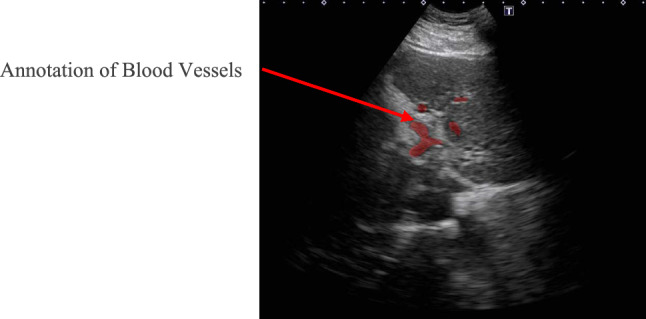
Example of annotation by the physician

In our previous research, we trained blood vessel segmentation models using ultrasound data of the liver from phantoms and pigs, but these models did not perform well in human liver recognition. Therefore, an AI model trained with patient US images was constructed in this study. To ensure that the acquisition conditions matched those during intraoperative navigation, only US images acquired using the robotic system from patients were used as training data. Specifically, all US images obtained through fan-shaped scans using the US probe attached to the robotic system’s end-effector were utilized. After being processed to ensure that no personal information could be identified, the US images were annotated by physicians to mark the locations of the blood vessels. An example of the annotation is shown in Fig. 3.

A total of 6828 US images were used to train an improved U-net model based on fivefold cross-validation. In this approach, the entire dataset was divided into five equal subsets, where one subset was used as the validation dataset, and the remaining four subsets were used as the training dataset. This process was repeated five times, changing the combination of training and validation datasets, ensuring that each image was used as validation data once. This method enhances the generalization performance of the model.

**Fig. 4:**
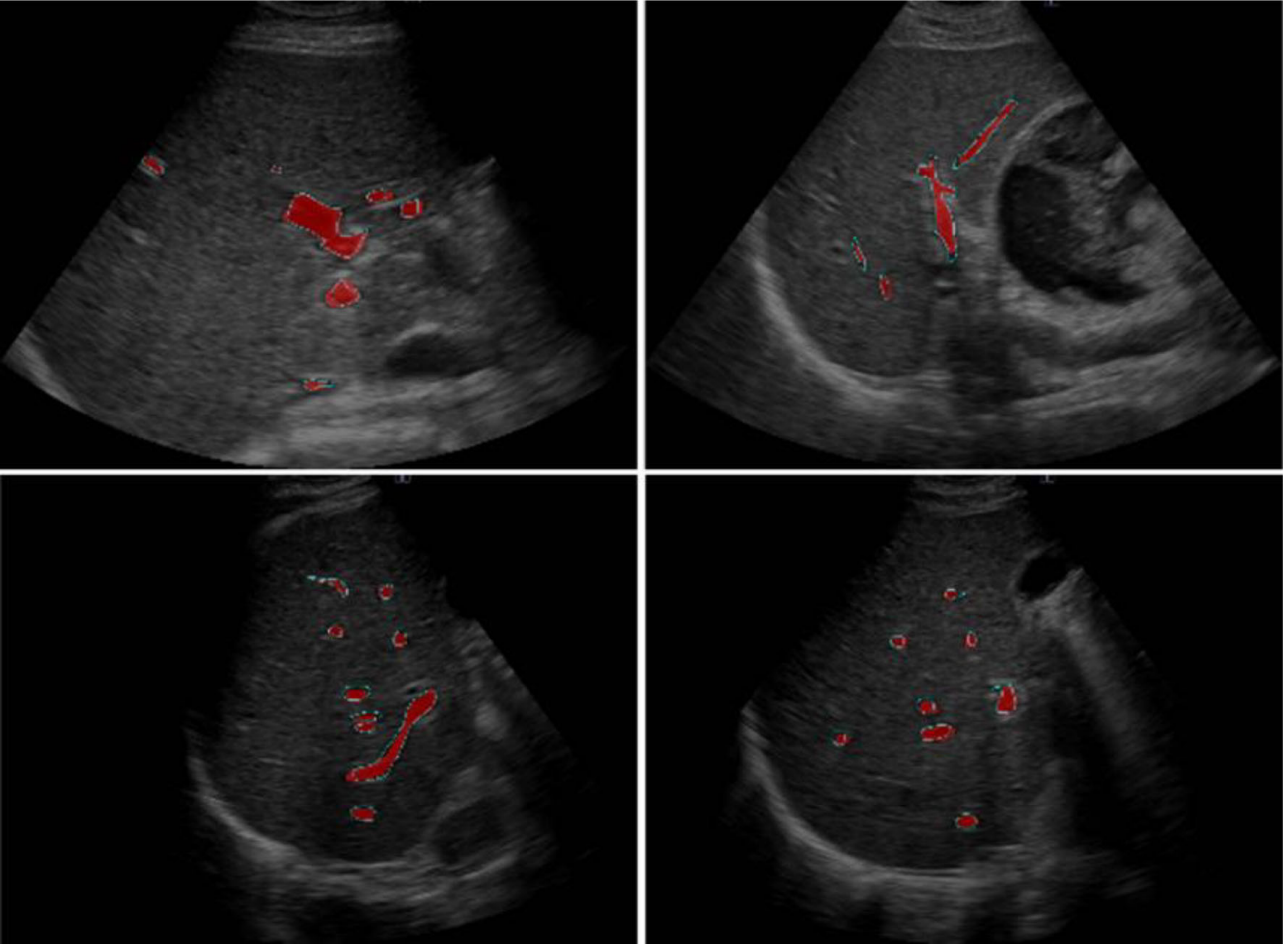
Segmentation results of the validation data

An example of the segmentation results for the validation data is shown in Fig. 4. The contours of the physician-annotated blood vessels are indicated by green lines, while the segmented regions produced by the AI model are highlighted in red. A high degree of agreement between the two regions was observed. For quantitative evaluation, the mean Dice coefficient of the entire validation dataset was calculated. The result was 0.87, indicating a high similarity between the annotated labels and the segmentation results. The details are presented in Table 1.

**Table 1:** Details of training data and DICE coefficient

Fold	Training set	Validation set	Mean dice
1	5462	1366	–
2	5457	1371	–
3	5462	1366	–
4	5462	1366	–
5	5463	1365	–
Total result	–	–	0.87

### Experiment and results

A clinical experiment using the robotic system equipped with the developed AI model was conducted on five adult male and female participants. This study was approved by the Waseda University Ethics Committee (Approval Number: 2024–328). The primary objective of the clinical experiment was to assess the accuracy of the fusion system, which synchronizes contrast-enhanced CT and US images through human testing, and to evaluate its potential utility in puncture navigation for further system improvement.

### Experimental approach

**Fig. 5:**
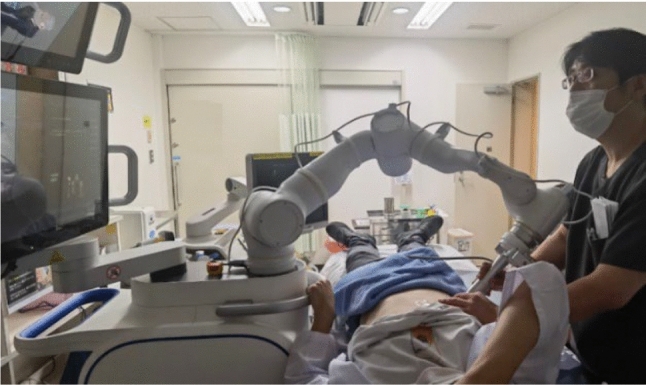
Experimental setup

**Fig. 6:**
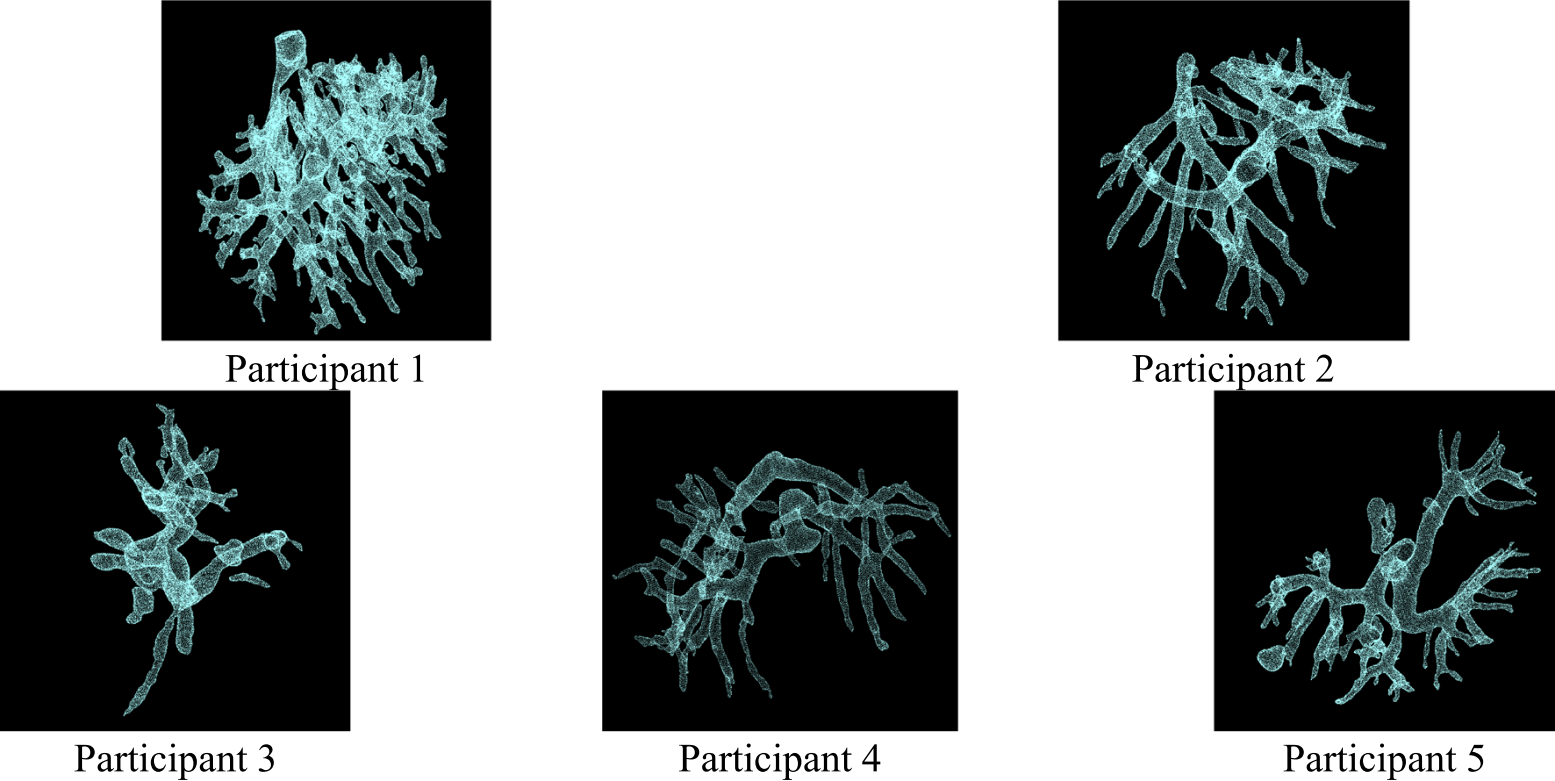
Three-dimensional CT point cloud of the participant constructed by preoperative planning

Figure 5 shows the clinical experiment setting. A 3D CT point cloud of the intrahepatic blood vessels was pre-constructed using preoperative planning software based on CT images with a slice thickness ranging from 1.0 to 2.5 mm. Figure 6 presents the constructed 3D CT point cloud. The CP classifications of each participant are shown in Table 2; participants 1 and 2 had no history of liver disease, while participants 3–5 were evaluated based on their CP classifications. The trial was conducted following a two-step procedure.

**Table 2:** The Child–Pugh classification of each subject

Participant no	Classification
1	No history
2	No history
3	CH-B
4	CH F3A1
5	LC CP-A

1. **Coarse Alignment of the Robotic System:** The robotic arm was positioned by the physician on the chest skin above the xiphoid process, where it could be confirmed in the US image. To ensure accurate coarse alignment with CT slices, the US probe cross-section was carefully placed parallel to the horizontal plane of the body.
2. **Fine Alignment of the Robotic System:** As shown in Fig. 5, the robotic arm was positioned by the physician on the intercostal skin where intrahepatic blood vessels were visible in the US image. To reduce interference from bony structures during fan-shaped scanning, the probe was aligned parallel to the ribs. After the participant was instructed to hold their breath, the robotic arm performed automated fan-shaped scanning, and US images were acquired. Using the acquired US images, 3D US–CT fusion was performed based on a feature-point registration method.

To evaluate variability in the results, multiple trials were conducted for each participant. For participants 1 and 2, who had no history of liver disease, the intrahepatic blood vessels were clearly visible in the US images, and five trials were performed for each. On the other hand, for participants 3–5, decreased visibility of blood vessels due to liver cirrhosis was observed. Since the proposed system relies on blood vessels visible in US images, it cannot be applied when blood vessels are undetectable. Therefore, for participants 3–5, results from three trials in which the blood vessels were deemed visible were obtained. Cases where the blood vessels were not visible are discussed as limitations of the system in the following section.

### Analytical approach

**Fig. 7:**
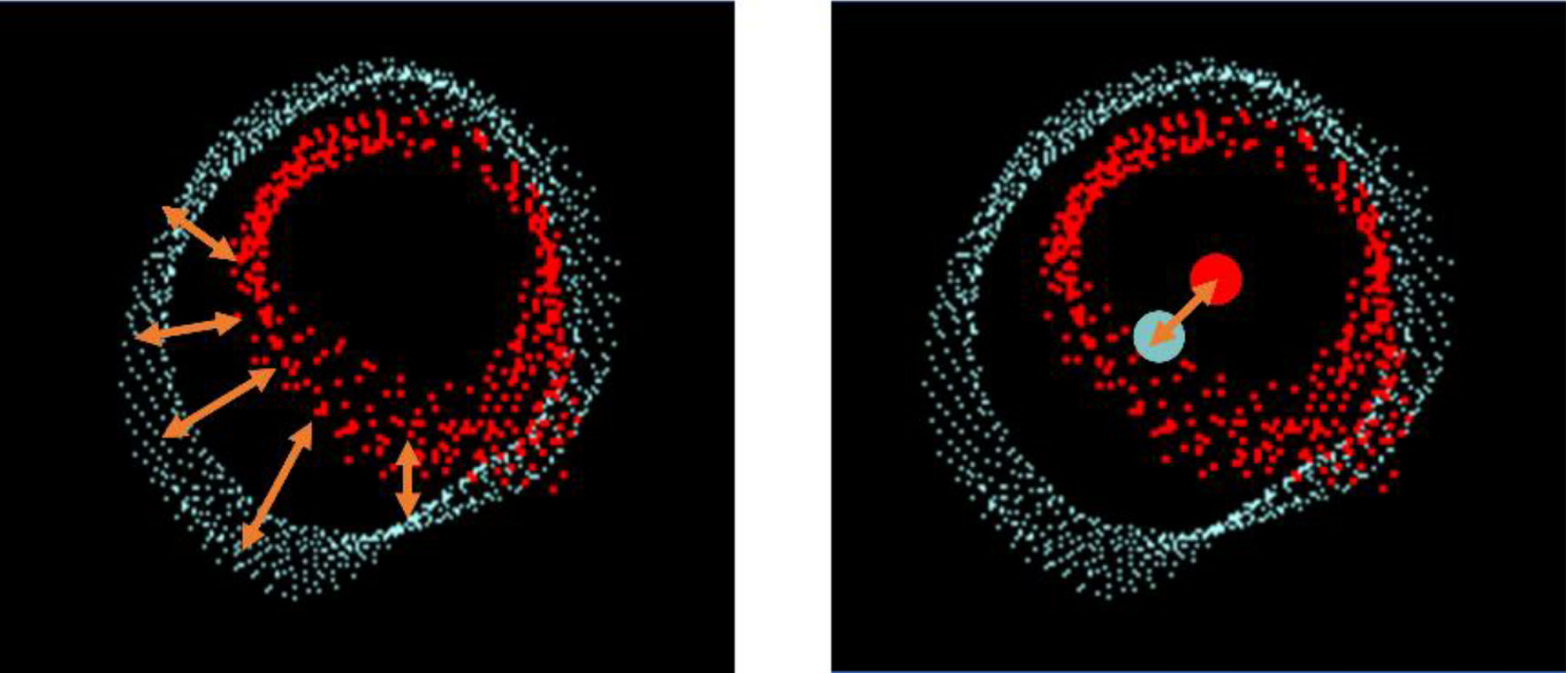
Issues in RMSE and the use of centroids

To quantitatively evaluate the degree of correspondence between the point clouds in 3D US–CT fusion, the Root-Mean-Square Error (RMSE) was first examined. RMSE is defined by Eq. (1):

1 $$\text{RSME}=\sqrt{\frac{1}{N}\sum_{i=1}^{N}{\left({d}_{i}\right)}^{2}}$$

where *N* represents the number of corresponding points, and *d*_*i*_ is the Euclidean distance between each corresponding point pair. RMSE is used as an index to measure the correspondence of point cloud assuming that the error distribution follows a Gaussian distribution [11]. Certain challenges may affect the evaluation of the blood vessel point cloud in this study. The blood vessel point cloud exhibits a hollow structure, as shown in Fig. 7, and a size difference between the point clouds due to modality differences was also observed. Consequently, calculating the RMSE for blood vessel point clouds from different modalities tends to amplify the impact of errors. Similar metrics, such as the Chamfer distance and Hausdorff distance, also show susceptibility to the influence of outliers in the US point cloud.

In fusion evaluation, the overall alignment of anatomical structures is considered more important than the positions of individual points. For blood vessel structures, the centerline of the blood vessels represents the overall structural position. Therefore, as shown in Fig. 7, the centroids of the two point clouds *P*_US_centor_ and *P*_CT_centor_, which approximate the centerlines of the vessels, were calculated, and the distance *D* between them was used as the evaluation metric. The centroid distance *D* is defined by Eqs. (2)–(4):

2 $${P}_{\text{US}\_\text{centor}}=\frac{1}{N}\sum_{i=1}^{N}{P}_{\rm US}^{i}$$

3 $${P}_{\text{CT}\_\text{centor}}=\frac{1}{N}\sum_{i=1}^{N}{P}_{\rm CT}^{i}$$

4 $$D=\sqrt{\sum_{k=1}^{3}{\left({P}_{\text{US}\_\text{center}}^{k}-{P}_{\text{CT}\_\text{center}}^{k}\right)}^{2}}$$

**Fig. 8:**
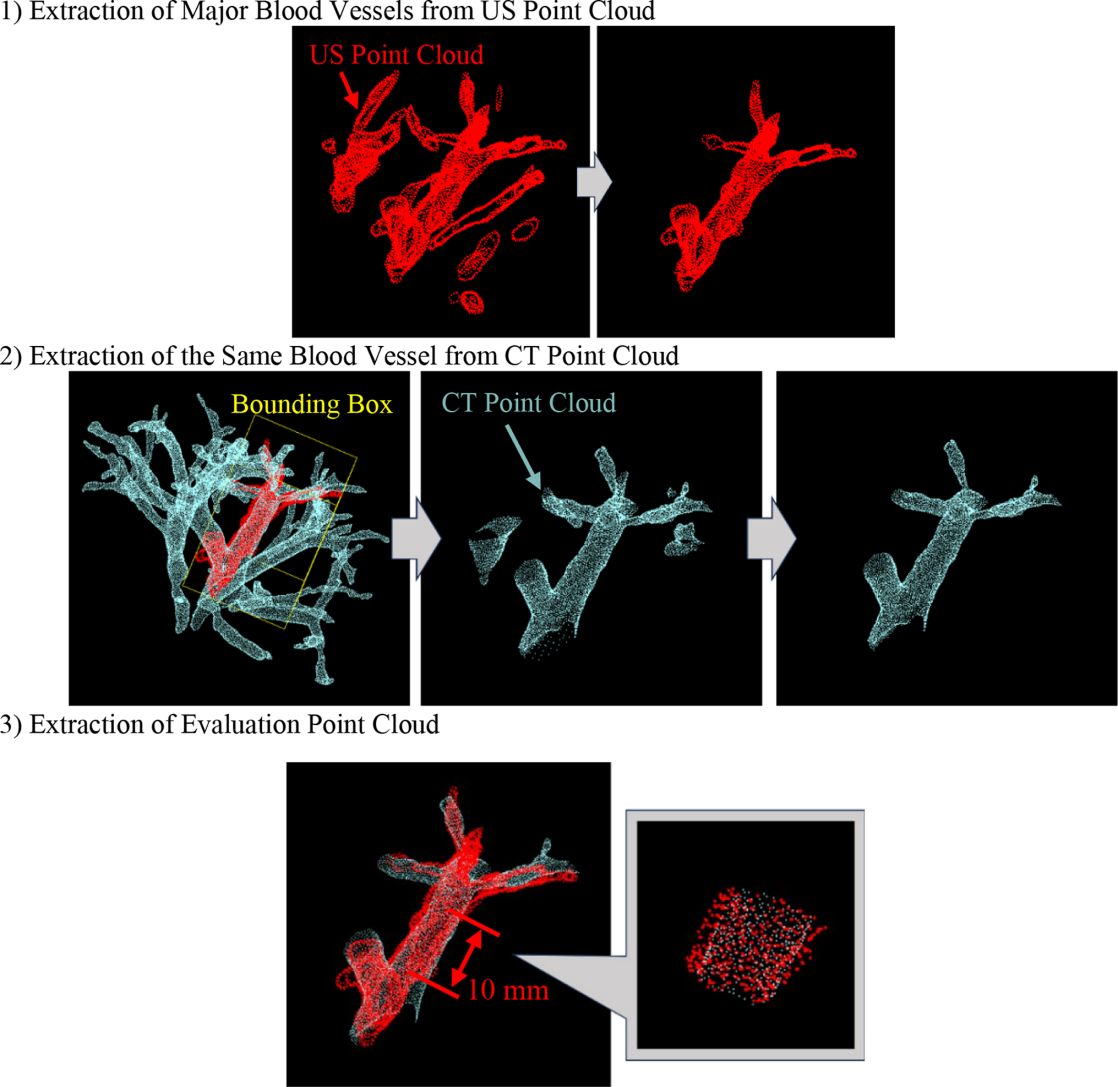
Analysis procedure of the 3D US–CT fusion results

The acquired point cloud underwent preprocessing to calculate the evaluation index. In this robotic system, blood vessel points clouds that appear in only one modality may exist due to differences in US image shadowing, contrast levels, or the timing of contrast-enhanced CT imaging. When calculating the centroid distance for the entire point cloud, the errors become large, making accurate evaluation difficult. Therefore, blood vessel point cloud for centroid distance calculation was extracted according to the following three steps, as summarized in Fig. 8:

1. **Extraction of the Primary Blood Vessel Point Cloud from the US Point Cloud:** To extract the largest blood vessel point cloud from multiple point cloud, the Density-Based Spatial Clustering of Applications with Noise (DBSCAN) algorithm was applied. The parameters were set to eps = 1.0 and "minimum points" = 10.
2. **Extraction of Corresponding CT Blood Vessel Point Cloud:** To extract the corresponding blood vessel region from the CT point cloud, a bounding box that minimally covered the primary US blood vessel point cloud was used to segment the CT point cloud. Subsequently, the same DBSCAN parameters were applied.
3. **Extraction of the Evaluation Point Cloud:** To remove the influence of point cloud present only in one modality, the point clouds were trimmed to retain only the common blood vessel branches observed in both modalities. A fixed cutting length was set to standardize the process.

### Evaluation

**Fig. 9:**
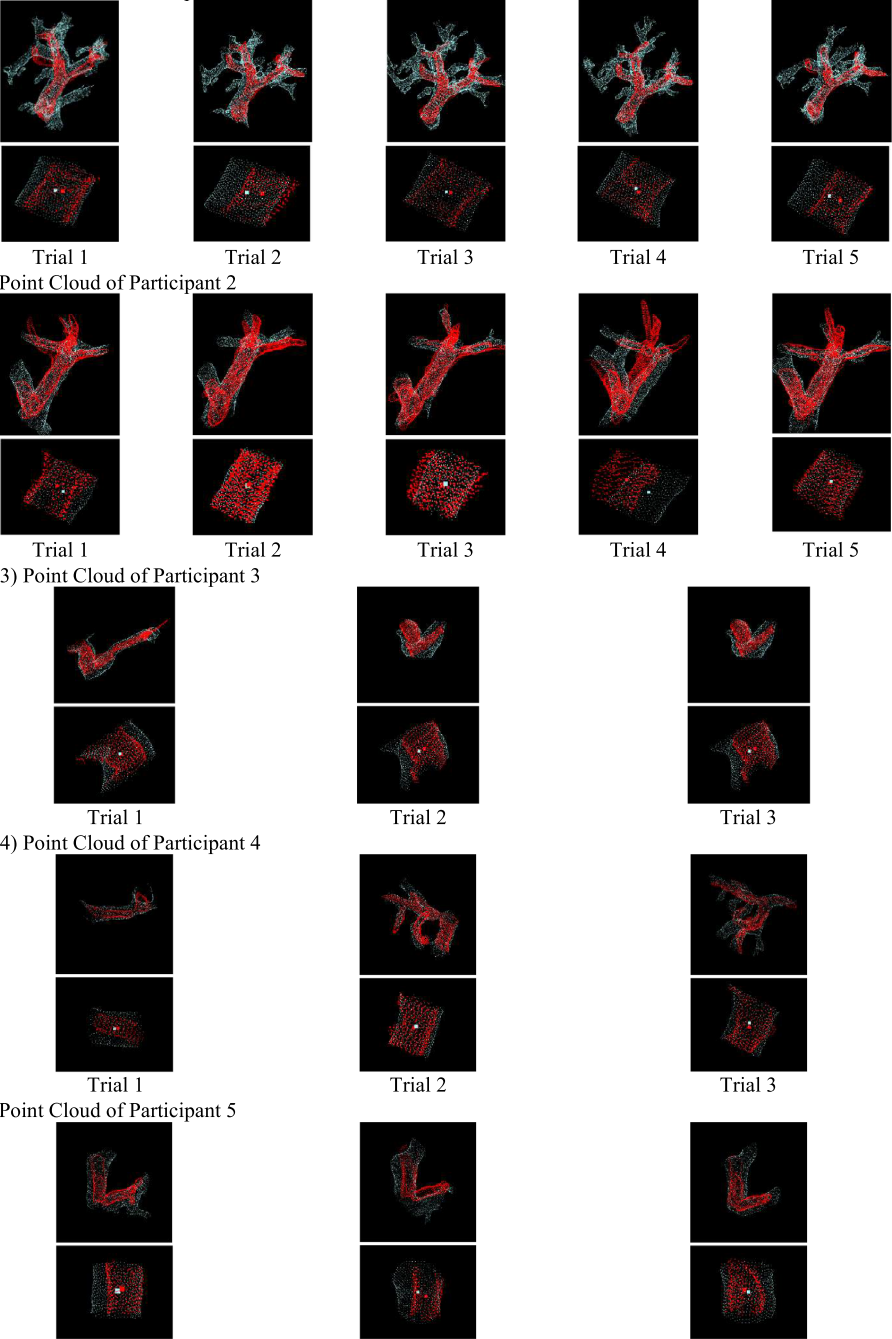
Extraction Results of Blood Vessel Point Clouds for All Participants

All point clouds obtained from each participant are shown in Fig. 9. The upper section of the figure shows the point clouds from each modality, trimmed using bounding boxes, while the lower section shows the extracted evaluation point clouds and centroids. The results of the 3D US–CT fusion demonstrated that the point clouds from the two modalities were in proximity, which was qualitatively confirmed. The distances between the centroids calculated from the evaluation point clouds are presented in Table 3. In this trial, the minimum centroid distance in the 3D US–CT fusion was 0.38 mm, the maximum was 4.81 mm, and the average across all 16 trials was 1.97 mm.

**Table 3:** Calculation results of centroid distance

Participant no	Centroid distance (mm)				
	Trial 1	Trial 2	Trial 3	Trial 4	Trial 5
1	1.68	3.06	1.37	0.87	2.28
2	2.88	0.61	1.13	4.81	2.47
3	0.38	1.68	1.86	-	-
4	1.94	1.44	1.14	-	-
5	2.00	2.40	3.34	-	-

The standard deviation of the centroid distances was 1.03 mm, with the average + 3*σ* range reaching 5.07 mm. Although all results in this study met the target accuracy, further improvement in the recognition accuracy of US point cloud is essential to ensure that the system consistently meets the target value over the long term.

The blood vessels used in this study had a complex structure, and appropriate preprocessing was performed due to differences in the point cloud shapes between the modalities. On the other hand, for deep veins such as the femoral and popliteal veins, which have relatively linear paths, methods have been proposed to approximate cross-sections of vessels in US images as ellipses to estimate the vessel course [12]. Based on these studies, further development of new methods is required to estimate the centerline of complex hepatic vessels and to evaluate the overall correspondence of point cloud.

### Limitations related to US image quality

**Fig. 10:**
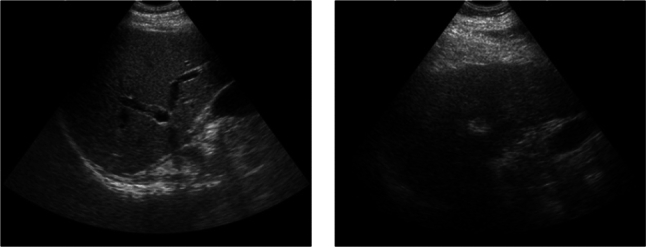
Examples of abundant and sparse blood vessels obtained during the clinical experiment

Figure 10 presents US images with abundant blood vessels and sparse blood vessels acquired during the clinical experiment. Due to subcutaneous fat, liver cirrhosis, or a history of prior surgery, fan-shaped scans are unable to obtain sufficient US blood vessels information or even fail to obtain any US blood vessels at sometimes, which will lead to the failure of US blood vessel point cloud reconstruction. Although research efforts have been made to improve the imaging performance of blood vessels in US images, unclear images are often excluded [13]. In addition, in robotic control studies using vascular segmentation, limitations still persist in situations where blood vessels are scarcely visible or within complex anatomical environments such as the abdomen [14]. In our study, even for patients with liver cirrhosis and low blood vessel visibility, shape reconstruction of partial vascular geometry was sufficient to achieve the target fusion accuracy, except in cases where no vessels were visible at all. However, in patients with thicker fat layers or more severe disease, it becomes difficult to manually locate vessels. Therefore, it is necessary to develop a system that can assess the abundance of blood vessels when the probe is initially placed and guide the probe to a position where vessels are plentiful.

### Safety of the robotic system

During the clinical experiment, no force exceeding 30 N was detected by the six-axis force sensor connected to the base of the probe, even during probe movement by the physician or the robotic arm, as well as due to abdominal motion caused by breathing. Since the emergency stop was never triggered, these results suggest that the system maintains safe contact with the human body.

## Conclusion

To reduce the burden on physicians and provide accurate and safe puncture in PRFA, a robotic puncture system utilizing 3D US–CT fusion was developed. In this study, the usefulness of the robotic system was evaluated through its pilot clinical experiment involving participants.

First, an improved U-net model based on fivefold cross-validation was constructed using patient US images as training data. High segmentation accuracy was confirmed in the validation data, and this model was implemented in the robotic system. Next, the accuracy of the 3D US–CT fusion was evaluated using five adult participants, both male and female. After the necessary preprocessing of the acquired point cloud, the centroid distances between the point clouds were calculated as an indicator of structural position. The minimum centroid distance in the 3D US–CT fusion was 0.38 mm, the maximum was 4.81 mm, and the average across all 16 trials was 1.97 mm, satisfying the required specification and indicating the system's potential utility.

As future work, further improvement in the recognition accuracy of US point cloud is required. Additionally, an evaluation of the robotic arm’s movement to the puncture position, which is the next process in the system, is expected. Since patients breathe freely during arm movement, it is necessary to develop methods to compensate for organ displacement caused by respiration and to address the influence of skeletal structures. Finally, new methods for evaluating the overall structural correspondence of complex hepatic blood vessels are planned to be developed.

## Declarations

### Use of language models

The language optimization of this paper was performed using a large language model (LLM).
